# Supplementary material for: Role of Ascorbic Acid in the Extraction and Quantification of Potato Polyphenol Oxidase Activity
Source: Foods. 2021 Oct 17;10(10):2486. doi: 10.3390/foods10102486 (PMC8535698; doi:10.3390/foods10102486)
Supplement: Supplementary file 1 [file foods-10-02486-s001.zip › foods-1339508-supplementary.pdf]

Supplementary Figure

Figure S1

(a)

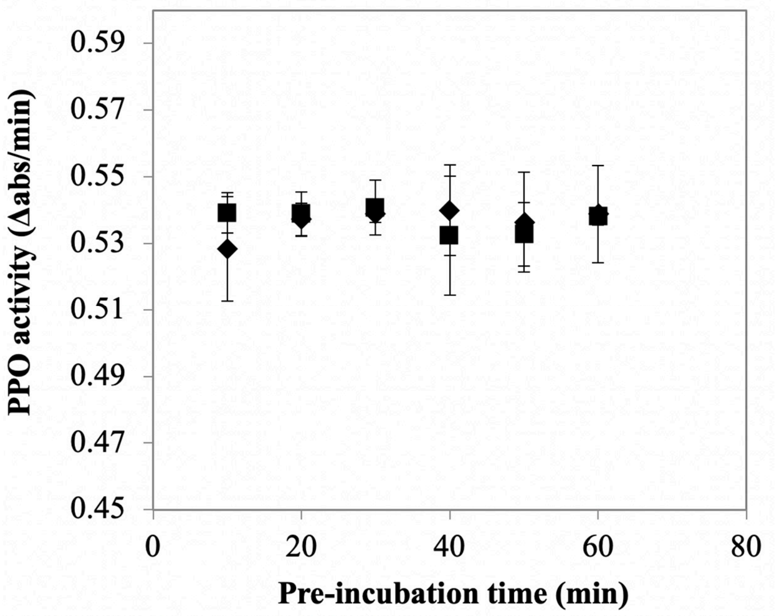

(b)

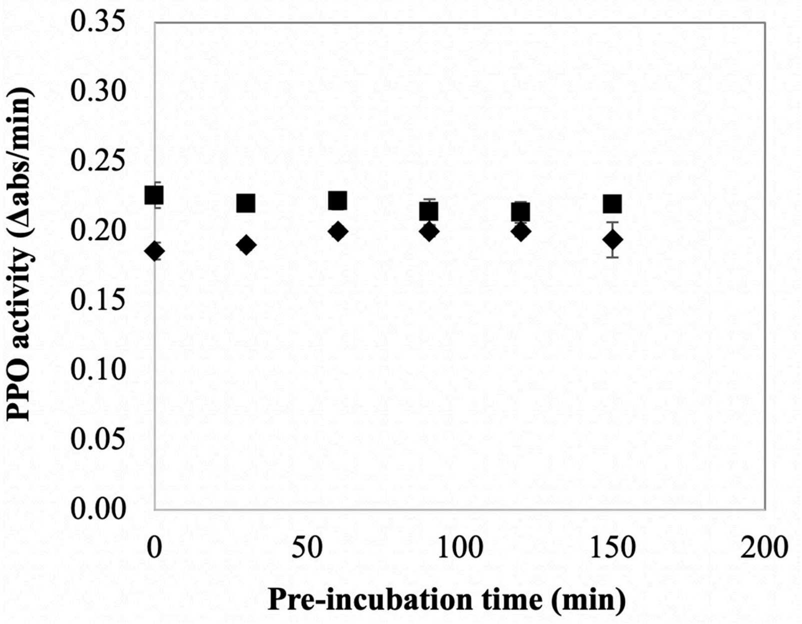

Figure S1. Effect of pre-incubating AA with semi-purified PPO preparation on subsequent enzyme activity measurements using (a) catechol and (b) CA as substrates. Semi-purified PPO

preparation was pre-incubated with (■) and without (◆) AA (AA at (a) 1 mM and (b) 10 mM when present) in 50 mM sodium phosphate buffer, pH 7.0, for different periods of time prior to initiating activity measurements by mixing 0.2 mL pre-incubation mixture (containing PPO and AA) with 2 mL buffered substrate solutions (final concentration 5 mM catechol or 1 mM CA). Prior to PPO activity measurements using catechol as a substrate, 1.25 unit/mL ascorbate oxidase was added to pre-incubation mixture (containing PPO and AA) to remove AA. Control experiments were treated identically but without AA. Values are means ± standard deviations from triplicate assays. Data points without visible error bars have standard deviations smaller than the designated symbols.

Supplementary Table

Table S1 Effect of pre-incubating AA with PPOE on subsequent PPO activity measurements using CA as a substrate. Enzyme extract (PPOE) was pre-incubated with 4 mM AA in 50 mM sodium phosphate buffer, pH 7.0, for 15 min prior to initiating activity measurements. PPO reactions were initiated by adding 0.2 mL pre-incubation mixture (containing PPOE and AA) to 2 mL buffered CA solution (final concentration 1 mM). Incubation time 0 min herein represents no AA pre-incubation with PPOE prior to the enzyme assay but AA was included simultaneously with the initiation of PPO reactions. Control experiments were treated identically but without AA. PPO was extracted from three individual potatoes. Values are means ± standard deviations from triplicate assays.

PPOE	incubation component	PPO activity (Δabs/min) pre-incubation time (min)	
		0	15
1	with buffer	0.086 ± 0.0028	0.088 ± 0.0035
	with AA	0.080 ± 0.0035	0.082 ± 0.0035
2	with buffer	0.134 ± 0.0069	0.134 ± 0.0035
	with AA	0.141 ± 0.0135	0.140 ± 0.0091
3	with buffer	0.112 ± 0.0125	0.120 ± 0.0000
	with AA	0.118 ± 0.0069	0.118 ± 0.0069
